# Engineering nitrogen-fixing microbiomes with waste-derived carbon sources: toward circular and resilient biofertilizer solution

**DOI:** 10.3389/fmicb.2025.1676616

**Published:** 2025-09-23

**Authors:** Nicolás Rodríguez-Romero, Juan Carlos Clavijo-Salinas, Julien Wist, Carlos Fernando Gutierrez, Daniel Uribe-Velez, Elaine Holmes, Janeth Sanabria

**Affiliations:** ^1^Agricultural Microbiology Research Group, Biotechnology Institute, National University of Colombia, Bogotá, Colombia; ^2^Environmental Microbiology and Biotechnology Laboratory, Faculty of Engineering, School of Natural Resources and Environmental Engineering, Universidad del Valle, Cali, Colombia; ^3^Australian National Phenome Centre and Centre for Computational and Systems Medicine, Health Futures Institute, Murdoch University, Perth, WA, Australia; ^4^Division of Digestive Diseases, Faculty of Medicine, Imperial College, London, United Kingdom; ^5^Institute of Global Health Innovation, Imperial College London, London, United Kingdom

**Keywords:** bioeconomy, waste valorization, targeted bioprospecting, self-assembled community, nitrogen

## Abstract

Microbiome engineering has emerged as a promising strategy to drive biotechnological developments across diverse fields. Microbiome-based fertilizers could significantly contribute to the gradual replacement of synthetic chemical fertilizers, potentially leading to substantial environmental and economic impacts. This study employed microbiome engineering to develop a self-assembled nitrogen-fixing microbial community utilizing carbon compounds from animal waste. This was achieved by enriching soil samples in bioreactors supplied with nitrogen via air pumping and fed with volatile fatty acids (VFAs) as the only carbon source. VFAs are the most common by-products of anaerobic waste fermentation. Results show a self-assembled community, dominated by *Sinirhodobacter* spp. (44.4%), *Aureimonas* spp. (17.7%), and *Taibaiella* spp. (12.4%), capable of fixing 2.7 times more nitrogen than the initial microbiome. During cultivation, inorganic nitrogen forms were detected in the supernatant at concentrations of up to 12.7 mg·L^−1^. Once the self-assembled community was inoculated in tomato plants, *Pseudomonas* spp. and *Exiguobacterium* spp. became the most abundant and significantly enhanced tomato plant growth in both hydroponic and soil-based systems. Plant height and yield were comparable to those achieved with conventional synthetic nitrogen fertilizers. This study shows the potential of this methodology for developing effective biofertilizers while promoting a circular economy strategy that transforms waste into high-value bioproducts. This approach, combined with the simplicity of the bioreactor system, offers a viable and sustainable solution for developing countries with limited technological resources, and materializes the One Health vision while simultaneously protecting the health of people, crops, and animals.

## 1 Introduction

In agriculture, numerous microbial products known as biofertilizers have been developed to promote plant growth (Shahwar et al., 2023). Most of these products are based on the traditional approach of isolating and formulating pure microbial cultures (Shahwar et al., 2023). However, their performance in field conditions is often inconsistent due to the limited adaptability of individual strains and the competition with native microbial communities, which results in low persistence in the soil environment (Roy, 2021). Recent advances in microbial ecology have driven the development of new microbiome-based biotechnologies across diverse fields, including human health, agriculture, environmental science, and the biotechnology industry (Naghavi and Samieipour, 2020; Lawson, 2021). In this context, microbiome engineering (ME) has emerged as a promising strategy to design self-assembled communities capable of performing key functions in a targeted manner (Lawson et al., 2019). ME may represent the next generation of biofertilizers, potentially overcoming the limitations of conventional single-strain formulations (Gutierrez et al., 2020).

Microbiome-based biofertilizers obtained through ME offer several advantages. First, they can provide a broader diversity of beneficial microorganisms, enhancing functional redundancy and soil ecosystem resilience (Gutierrez et al., 2020). Additionally, using microbiomes instead of single strains allows for the exploitation of synergistic interactions among different microorganisms (Lawson, 2021), which can improve adaptability to different crops, soils, and environmental conditions.

One of the most promising targets for ME in agriculture is biological nitrogen fixation (BNF). BNF is the process by which certain prokaryotes, known as nitrogen-fixers, convert atmospheric nitrogen gas (N₂) into ammonia (NH₃), a form that plants and other organisms can readily assimilate (Myrold, 2021). Nitrogen is frequently the most limiting nutrient for crop production, and the widespread use of synthetic nitrogen fertilizers has led to a severe increase in greenhouse gasses and eutrophication (Menegat et al., 2022; Schulte-Uebbing et al., 2022). While BNF-based biofertilizers offer a sustainable alternative, their large-scale implementation remains challenged by issues of efficiency and production cost (Bonilla Buitrago et al., 2021). The commercial production of nitrogen-fixing biofertilizers, such as *Rhizobium* spp., *Azospirillum* spp., or *Azotobacter* spp., typically relies on conventional carbon sources like sucrose, malic acid, mannitol, or glucose, which can account for up to 54% of variable production costs (Amaresan et al., 2023). This highlights a critical opportunity to reduce costs using alternative waste-derived carbon sources, particularly volatile fatty acids (VFAs).

Globally, around 55 billion tons of animal waste are generated annually (Girotto and Cossu, 2017). Anaerobic digestion has become a widely adopted strategy to manage and valorize this waste, mainly for biogas production (Clavijo-Salinas et al., 2020). More recently, attention has shifted toward recovering value-added organic compounds, such as VFAs, from these processes (Koubaa, 2024). It is estimated that waste transformation could yield 9.15 Mt. of acetic acid, 5.39 Mt. of butyric acid, and 6.47 Mt. of propionic acid each year (Atasoy et al., 2018). These VFAs have already been used as carbon sources to produce diverse bioproducts, including polyhydroxyalkanoates (PHAs) (Ospina-Betancourth et al., 2022), as well as biofuels, biogas, and hydrogen, among others (Agnihotri et al., 2022). However, the direct use of VFAs to feed nitrogen-fixing bacteria is not widely documented in the current scientific literature. These bacteria typically require specific carbon sources and particular conditions to carry out biological nitrogen fixation.

In this study, we applied nutrient availability as a selective condition to shape the composition of a self-assembled nitrogen-fixing community (SANF). Atmospheric N₂ was used as the sole nitrogen source, and a synthetic VFAs mix as the carbon source. The engineered SANF was fed with a mixture of volatile fatty acids (VFAs) derived from the anaerobic digestion of swine waste, serving as the main carbon source. We then evaluated this SANF consortium for its potential use as a biofertilizer in tomato plants (*Solanum lycopersicum*).

## 2 Materials and methods

### 2.1 Microbial inoculum

To ensure high microbial diversity, the initial inoculum was obtained from the rhizosphere soil of cassava (*Manihot esculenta*), plantain (*Musa paradisiaca*), aloe (*Aloe vera*), and brachiaria grass (*Brachiaria decumbens*) collected from the “Cañabravas” farm, located in the San José village, Darién municipality, Valle del Cauca, Colombia (3°53′12.9”N, 76°22′9.2”W). This is a family-run farm with no history of synthetic nitrogen fertilizer application. Three plants from each species were uprooted, and the bulk soil was vigorously shaken off. Young roots with root hairs were selected, cut, and stored in plastic bags at 4°C for transport to the laboratory. For each plant species, 100 g of roots were weighed. Roots from the four species were pooled in a sterile 1 L flask, filled halfway with 0.85% NaCl, and supplemented with 0.01% Tween 80. The mixture was agitated at 300 rpm for 90 min to detach rhizosphere soil and rhizoplane-associated bacteria. Then, 50 mL of the liquid phase was centrifuged at 150 × g for 10 min. The supernatant was filtered through a 1 mm pore-size filter into a sterile 50 mL plastic tube. This filtrate was used as the starting inoculum for the microbiome engineering process.

### 2.2 Microbiome engineering – enrichment phase

**Figure 1:**
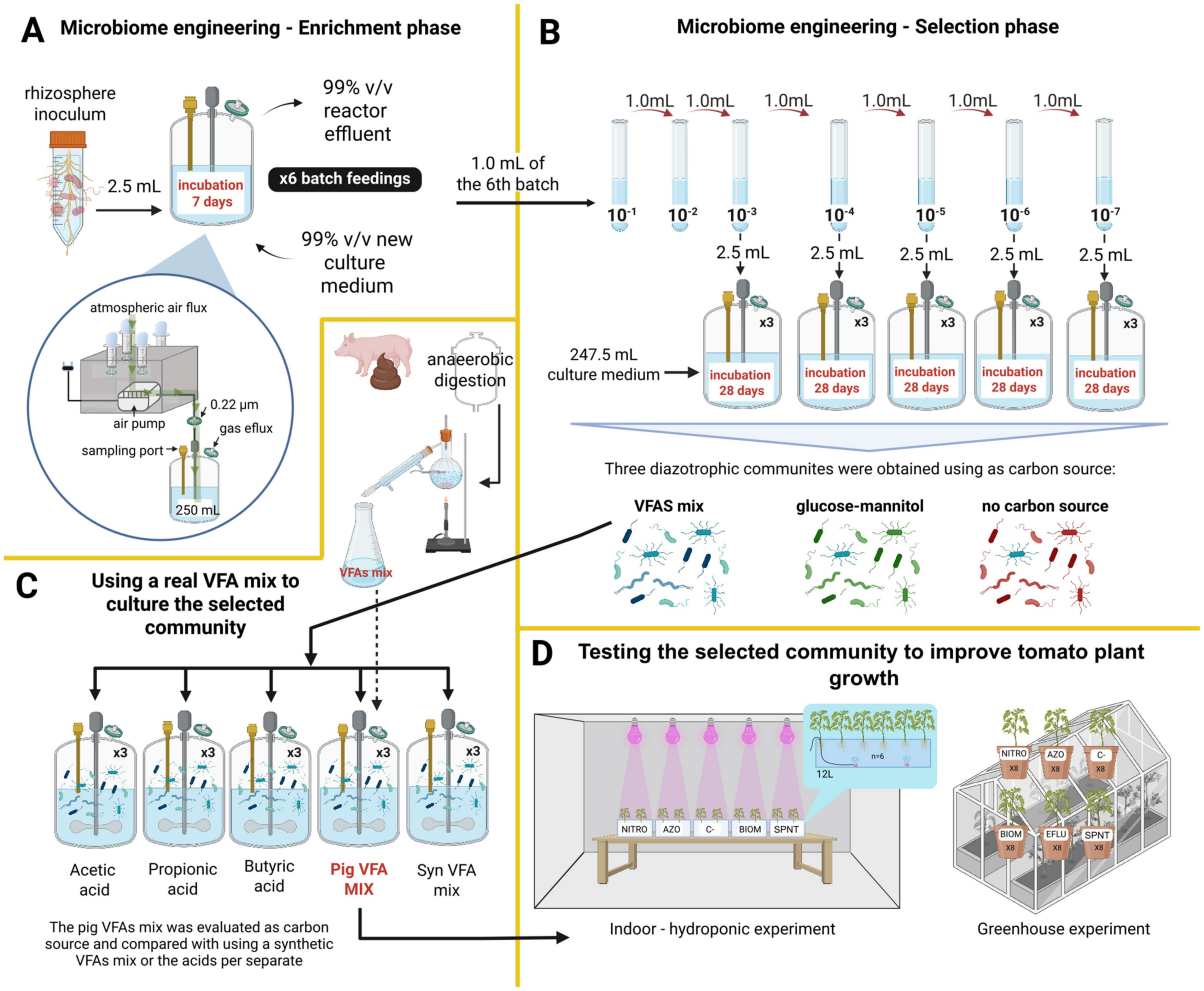
Experimental setup. **(A)** Enrichment phase and bioreactor setup. This phase is aimed to gradually increasing the presence of nitrogen-fixing microorganisms while reducing the presence of non-fixers. Enrichments were performed using three different carbon sources: a synthetic mixture of volatile fatty acids (VFAs), a glucose–mannitol mixture, and a control with no added carbon source. **(B)** Selection phase. The objective of this phase was to simplify the enriched communities obtained in the enrichment phase. The selection was also carried out using the same three carbon sources. **(C)** Evaluation of VFAs from pig manure as a carbon source for nitrogen fixation. The selected community from the selection phase was assessed using a mixture of VFAs derived from pig manure. Its growth and nitrogen-fixing capacity were compared with those obtained using individual VFAs and a synthetic VFA mixture. **(D)** Evaluation of the biofertilizer potential. The community fed with pig-manure-derived VFAs was tested as a biofertilizer by inoculating tomato plants and evaluating their growth response. VFAs: volatile fatty acids. See the text for further details.

This phase aims to gradually increase the presence of N-fixers while non-fixers are progressively eliminated. Nine 500 mL total volume vessels were prepared, each containing 247.5 mL of culture medium and 2.5 mL of a microbial suspension obtained from rhizosphere soil. Three of the bioreactors were supplied with a synthetic mixture of volatile fatty acids (VFAs) as the sole carbon source (7.45 g/L of acetic acid, 2.25 g/L of propionic acid, and 1.85 g/L of butyric acid), supplemented with N-free basal (NFB) medium. The concentration of the synthetic mixture of VFAs was based on the reported proportion of these acids in a distilled mixture of VFAs from pig manure (Huang et al., 2016). Another three bioreactors received a mixture of 8 g/L glucose and 8 g/L mannitol as a carbon source, also with the NFB medium. Three bioreactors received only the NFB medium. NFB medium composition: 0.5 g/L K₂HPO₄, 0.1 g/L NaCl, 0.2 g/L MgSO₄, 2 g/L KOH, 0.02 g/L CaCl₂, 2 mg/L Na₂MoO₄·2H₂O, 2.35 mg/L MnSO₄, 2.8 mg/L H₃BO₃, 0.08 mg/L CuSO₄·5H₂O, and 0.024 mg/L ZnSO₄·7H₂O, 33.03 mg/L FeSO₄·7H₂O and 22.24 mg/L of Na₂EDTA·2H₂O. The pH of the medium was adjusted to 7. Filtered air was used as the source of nitrogen and oxygen, passed through a cotton plug (to prevent particle entry) and a 0.22 μm filter (Figure 1A). All nine bioreactors were operated in fed-batch mode with 7-day cycles repeated six times. At the start of each 7-day cycle, 99% of the reactor contents were replaced with fresh medium (Figure 1A). At the end of each cycle, aliquots were collected to measure optical density at 600 nm (Hoefer SP-2001) and pH (Hanna Instruments HI-98160).

### 2.3 Microbiome engineering – selection phase

To obtain the best assembly of microorganisms for the self-assembled nitrogen-fixing community (SANF), a selection phase was conducted. At the end of the enrichment process (6th batch feeding), 1 mL of culture from each of the 9 reactors was collected and subjected to serial dilutions. Dilutions ranging from 10^−3^ to 10^−7^ for each of the three carbon source treatments were used to reconstitute new bioreactors by adding 2.5 mL of the diluted culture to 247.5 mL of fresh NFB medium. Three replicates were prepared for each dilution. The resulting 15 bioreactors per carbon source were incubated under constant agitation for 28 days at room temperature with a continuous air flow of 1 L/min (Figure 1B). The bioreactors with NFB + AGVs were named as A-3, A-4, A-5, A-6, and A-7. The bioreactors with NFB + glucose-mannitol were named as G-3, G-4, G-5, G-6, and G-7. The bioreactors only with NFB medium were named as S-3, S-4, S-5, S-6, and S-7. Every 7 days, aliquots were taken to measure optical density at 600 nm (Hoefer SP-2001), pH (Hanna Instruments HI-98160), total nitrogen (Hanna Instruments HI93767A-50), and VFAs concentrations. Sterile distilled water was added as needed to maintain an effective working volume of 250 mL throughout the experiment. VFAs were analyzed by gas chromatography (Perkin Elmer Clarus 590) using an Elite FFAP column (30 m × 0.25 mm ID × 0.25 μm DF) and a flame ionization detector. Nitrogen was used as the carrier gas at 15 psi, with a split ratio of 120 mL/min. The oven, injector, and detector temperatures were maintained at 140°C, 250°C, and 250°C, respectively. Samples for VFA analysis were acidified with 37% HCl (Honeywell Fluka, Austria), filtered using 0.45 μm PTFE membranes, and stored at −20°C until analysis. A 10 mM standard VFA mix (Sigma-Aldrich, United States) was used to construct the calibration curve, including acetic acid, propionic acid, isobutyric acid, butyric acid, isovaleric acid, valeric acid, 4-methylvaleric acid, hexanoic acid, and heptanoic acid. The reactor with the highest amount of fixed nitrogen using VFAs was selected as the SANF community and was used for further experiments.

### 2.4 Evaluation of a VFAs mixture derived from swine manure as a carbon source

In this experimental phase, a VFAs mixture derived from swine manure was evaluated as a carbon source for the SANF community and compared against other carbon sources (Figure 1C). The tested carbon sources were: (1) Pig VFA mix: acetic acid (463.10 mg/L), propionic acid (108.33 mg/L), butyric acid (123.40 mg/L), isobutyric acid (40.63 mg/L), valeric acid (29.29 mg/L), and isovaleric acid (64.16 mg/L); (2) Synthetic VFA mix: acetic acid (600 mg/L), propionic acid (100 mg/L), and butyric acid (130 mg/L); (3) acetic acid (830 mg/L); (4) propionic acid (830 mg/L); and (5) butyric acid (830 mg/L). For the 2, 3, 4, and 5, the concentrations of VFAs were adjusted to have the same total VFAs concentration as the pig VFA mix. Each carbon source treatment was supplemented with the NFB medium. For each 500 mL reactor, 247.5 mL culture medium and 2.5 mL of the inoculum with the highest nitrogen fixation performance from the selection phase were added. The bioreactors were operated in batch mode for 28 days under complete mixing and continuous airflow to provide nitrogen and oxygen, for enrichment of aerotolerant bacteria. Samples were collected weekly to measure pH, optical density at 600 nm (OD₆₀₀), total nitrogen, and VFA concentrations. Sterile distilled water was added as needed to maintain an effective working volume of 250 mL throughout the experiment.

### 2.5 Evaluation of the biofertilizer potential of the SANF community on tomato plants grown in hydroponic and soil systems

For the hydroponic experiment, a floating raft system (Son et al., 2020; Figure 1D) was used to evaluate the effect of both the supernatant and biomass obtained from the SANF fed with a mixture of pig-derived VFAs. Tomato seeds of the UNAPAL-Maravilla variety (Estrada Salazar et al., 2004) were used. Seeds were surface disinfected and germinated in Petri dishes in the dark for 4 days at 25°C and subsequently transplanted into the hydroponic system. Each tank had a working volume of 12 L filled with tap water. Oxygenation of the nutrient solution was achieved using an air pump providing a constant flow, maintaining a dissolved oxygen level of 2%. Growth room conditions were maintained at an average temperature of 26 ± 3°C, 70 ± 4% relative humidity, and a 12-h photo period supplied by plant growing LED lights.

A completely randomized design was used with five treatments. Each tank corresponded to a different treatment, and six plants were used per tank (Figure 1E). The first treatment consisted of the application of microbial biomass (BIOM) obtained from the effluent of the reactor fed with pig-derived volatile fatty acids (VFAs). The effluent was centrifuged at 5082 x *g* for 30 min, and the pellet was resuspended in sterile 0.85% NaCl solution. The suspension was adjusted to an optical density of 0.3 at 600 nm (Hoefer Vision SP-2001). A volume of 10 mL of this suspension was applied weekly to the hydroponic tank for three consecutive weeks. The second treatment (SPNT) used the supernatant from the same effluent, collected after centrifugation, sterilized, and analyzed for total nitrogen content (9.8 mg/L, Hanna Instruments HI93767A-50). A volume of 2.57 L was added to each tank and adjusted to 12 L with tap water to match the nitrogen content of the Hoagland solution (0.21 mg/mL). The third treatment (AZO) consisted of the application of a commercial biofertilizer (Dimazos®, Colombia) formulated with *Azotobacter chroococcum* and *Azospirillum* sp. Following the dosage recommended by the manufacturer, 10 mL of the product was applied weekly for 3 weeks. The positive control (NITRO) received full-strength Hoagland solution once at the beginning of the experiment (Taiz et al., 2022), while the negative control (C-) used a nitrogen-free Hoagland solution (Gutiérrez et al., 2022). All treatments received the same macro- and micronutrient supply from a modified Hoagland solution without nitrogen.

For the greenhouse experiment, tomato seedlings (UNAPAL-Maravilla) were transplanted into 3 kg soil bags, using soil collected from the upper 20 cm of a forest under natural restoration in the experimental station on the University of Valle campus (3°22′23”N, 76°31′51”W; Cali, Colombia). The experiment was conducted under controlled conditions (23 ± 4°C, 70 ± 3% relative humidity, 12 h photoperiod). A completely randomized design was used with six treatments. For each treatment, 8 replicates were used, each consisting of one tomato plant. The treatments mirrored those in the hydroponic setup, with application volumes adjusted to meet a target of 12 g nitrogen per plant where applicable. The BIOM and SPNT treatments were prepared as described before, but different amounts of microbial suspension were applied to the soil. For BIOM, a volume of 10 mL of the product was applied as a drench to each plant weekly for 4 weeks. For the SOBRE treatment, a volume of 662 mL was applied to each plant weekly for 4 weeks. For the AZOTO treatment, 7.5 mL was applied per plant as a drench weekly for 4 weeks, following the manufacturer’s recommended dosage. For the NITRO treatment, nitrogen fertilization was provided using full-strength Hoagland solution. A volume of 12.37 mL was applied per plant weekly for 4 weeks. This amount was calculated to deliver 12 g of nitrogen per plant, considering the crop’s nutritional needs and the soil’s initial nitrogen content. The C- treatment consisted of a nitrogen-free Hoagland solution, prepared as described before. A volume of 12.37 mL was applied per plant weekly for 4 weeks. Lastly, for the EFLU treatment, the reactor effluent was directly adjusted to an optical density of 0.3 at 600 nm and applied as a 10 mL drench per plant weekly for 4 weeks. In all treatments, macro- and micronutrients were provided using a modified Hoagland solution without nitrogen as described.

For the plants grown in hydroponics, 45 days after transplanting (vegetative growth phase), agronomic variables such as stem length, number of leaves, root length, total fresh weight, and total chlorophyll were measured. Additionally, the composition of the rhizosphere microbiome was determined (Section 2.7). For the plants grown in the greenhouse, plant height and number of leaves were measured weekly until day 70 after transplanting (DAT). Fresh fruit weight production was measured for each treatment from day 45 until day 90 after transplanting. Additionally, the composition of the rhizosphere microbiome was determined (Section 2.7) at 35 days after transplanting (vegetative growth phase).

### 2.6 Metataxonomic analysis of different microbiomes

Targeted sequencing of the 16S rRNA gene was performed to determine the taxonomic composition at different stages of the experimental setup. Specifically, the taxonomic composition was analyzed for the initial rhizosphere inoculum (IR), serial dilutions from the selection phase fed with VFAs (V-3 to V-6), reactors fed with synthetic and swine-derived VFA mixtures, and the rhizosphere of the AZO, BIOM, and C- treatments in both the hydroponic and greenhouse experiments.

For the reactor samples, 50 mL of effluent was centrifuged at 10,000 × g for 30 min (UNIVERSAL 320 - Hettich). The supernatant was discarded, and the resulting pellet was used as the starting material for DNA extraction. For hydroponically grown plants and the plants grown in soil, rhizosphere microorganisms were collected following the methodology described in 2.1. The rhizosphere soil suspension was centrifuged at 10,000 × g for 30 min. The pellet was used for DNA extraction. DNA was extracted using the NucleoSpin Soil kit (Macherey–Nagel GmbH, Germany), following the manufacturer’s instructions (lysis solution SL1). DNA quantity and quality were assessed by agarose gel electrophoresis and spectrophotometry using a Nanodrop 2000 (Thermo Scientific, United States). The V3–V4 regions of the 16S rRNA gene (primers 341F and 805R) were sequenced by MR. DNA Lab (Molecular Research LP Co, TX, USA) using an Illumina MiSeq platform.

Sequencing data were analyzed using QIIME2 v2023.9^1^ (Bolyen et al., 2019). Noise removal, sequence dereplication, and chimera filtering by consensus were performed using DADA2 (Callahan et al., 2016) within the QIIME2 pipeline. Taxonomic assignment of amplicon sequence variants (ASVs) was conducted using the fit-classifier-naïve-Bayes plugin against the SILVA 138 database. Visualization of taxonomic abundances and diversity indices was performed using the Phyloseq package (McMurdie and Holmes, 2013) in R (R Core Team, 2022).

### 2.7 Statistical analysis

For the monitoring variables of the bioreactors, an analysis of variance (ANOVA) was performed (*p* < 0.05). The assumptions of normality were tested using the Shapiro–Wilk test (*p* < 0.05), and homogeneity of variances was assessed using Bartlett’s test (*p* < 0.05). Multiple means comparisons were carried out using Tukey’s test (*p* < 0.05). Comparisons between treatments with a non-inoculated control or day 0 were carried out using the Dunnett test (*p* < 0.05). To evaluate the relationships between selection conditions and microbial community dynamics, a Pearson correlation analysis was performed between the measured physicochemical parameters (pH, VFAs consumption, and total nitrogen) and the relative abundance of the most abundant taxa detected during the selection phase. All analyses were performed using R software (R Core Team, 2022).

## 3 Results

### 3.1 The SANF community, through the enrichment and selection process, is capable of fixing nitrogen and consuming VFAs as a carbon source

**Figure 2:**
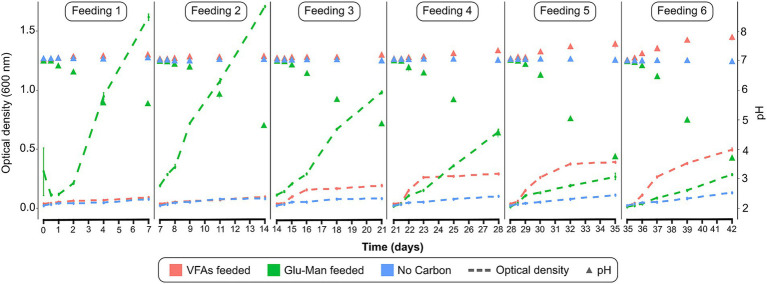
Microbial growth in the enrichment phase. Optical density (dashed lines) and pH (triangles) for each of the six successive feedings. VFAs: volatile fatty acids; Glu-Man: glucose and mannitol mixture *n* = 3.

To selectively enrich nitrogen-fixing microorganisms, a rhizosphere inoculum was incubated for 42 days in bioreactors supplied with either VFAs, glucose-mannitol, or without carbon sources. The cultures were maintained in NFB medium, with continuous aeration via filtered air. To sustain microbial activity, the semi-batch feeding process, in which 99% of the medium was replaced with fresh medium every 7 days, was employed. The results show that at the beginning of the enrichment phase (Figure 2), lower growth was observed when VFAs were used as the carbon source compared to glucose and mannitol. However, by the end of feedings 5 and 6, higher growth was observed with VFAs (Figure 2). VFA consumption led to an increase in pH, whereas glucose-mannitol metabolism resulted in a pH decrease (Figure 2).

**Figure 3:**
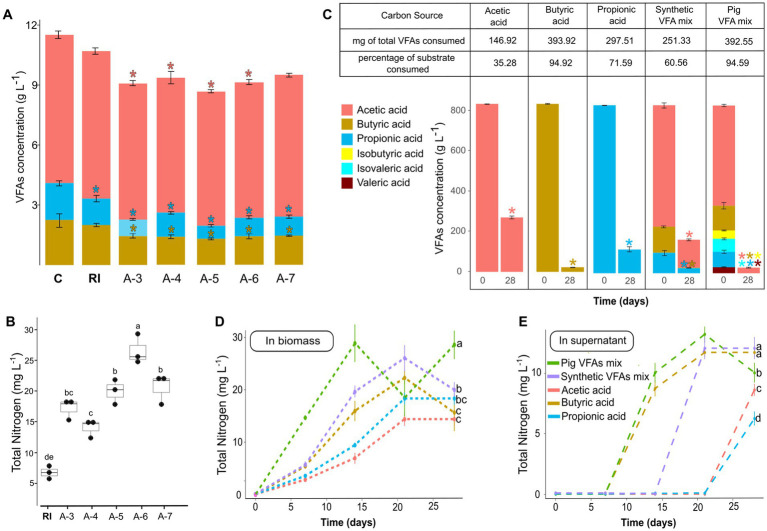
VFAs consumption **(A)** and total nitrogen production **(B)** during the selection phase measured after 28 days of reactor operation. Asterisks in **A** indicate significant differences in VFA concentration compared to the uninoculated control (ANOVA-Dunnett, *p* < 0.05). Different letters in **B**, indicate significant differences in total nitrogen (ANOVA-Tukey, *p* < 0.05). *n* = 3. C: uninoculated control, RI: rhizosphere inoculum, reactors inoculated with the dilutions from 10^−3^ to 10^−7^ were named as A-3, A-4, A-5, A-6, and A-7, respectively. VFAs consumption **(C)** and total nitrogen in the biomass **(D)** and in the supernatant **(E)** of bioreactors fed with VFAs mix from pig manure after 28 days of operation. Asterisks indicate significant differences in VFA concentration compared to day 0 (ANOVA-Dunnett, *p* < 0.05). Different letters indicate statistically significant differences on day 28 (ANOVA-Tukey, *p* < 0.05).

For the selection phase, aliquots from each reactor at the end of the enrichment phase were serially diluted and used to inoculate new bioreactors under the same carbon source conditions. These bioreactors were incubated for 28 days. On day 28, a significant reduction in butyric and propionic acid concentrations was observed across all reactors, and a significant reduction in the concentration of acetic acid was observed in A-3, A-4, A-5, and A-6 bioreactors (Figure 3A) compared to an uninoculated control (C). Additionally, the use of VFAs in the A-6 reactors resulted in a significantly higher total nitrogen concentration compared to the inoculum without any enrichment or selection process (RI) (Figure 3B). In contrast, the use of glucose-mannitol during the selection phase resulted in significantly lower total nitrogen levels compared to the initial rhizosphere inoculum (RI) (Supplementary Figure S1).

### 3.2 The SANF community is capable of fixing nitrogen using a mixture of VFAs from waste

To test the applicability of a real VFA mixture from waste, we fed the self-assembled community with a VFAs mix from anaerobic digestion of pig manure and compared the results with those obtained using a synthetic mixture and individual VFAs. After 28 days of batch culture, significantly higher growth was observed when using a VFAs mix, whether synthetic or derived from pig manure (Supplementary Figure S2). The use of butyric acid alone also led to significantly greater growth compared to using either acetic acid or propionic acid alone (Supplementary Figure S2).

All treatments began with the same total VFA concentration, and differential consumption was observed. Over 90% of the VFAs were consumed in the pig manure-derived mixture and the butyric acid treatments (Figure 3C). The lowest consumption was observed for acetic acid, with only 35.28% of the initial amount being utilized. The effluent from the bioreactors was centrifuged, and total nitrogen was measured in both the biomass and the supernatant. A peak in total nitrogen within the biomass was observed in the treatment fed with the pig manure-derived VFA mixture (Figure 3D). This peak coincides with the onset of the stationary phase at day 14 of operation. Similarly, nitrogen was detected in the supernatant starting on day 14 for the treatments with butyric acid and pig manure-derived VFAs, and on day 21 for the synthetic VFA mixture (Figure 3E); this also coincides with the onset of the stationary phase.

### 3.3 The SANF community inoculated in tomato plants promotes growth and yield equivalent to that obtained with synthetic nitrogen fertilization

**Figure 4:**
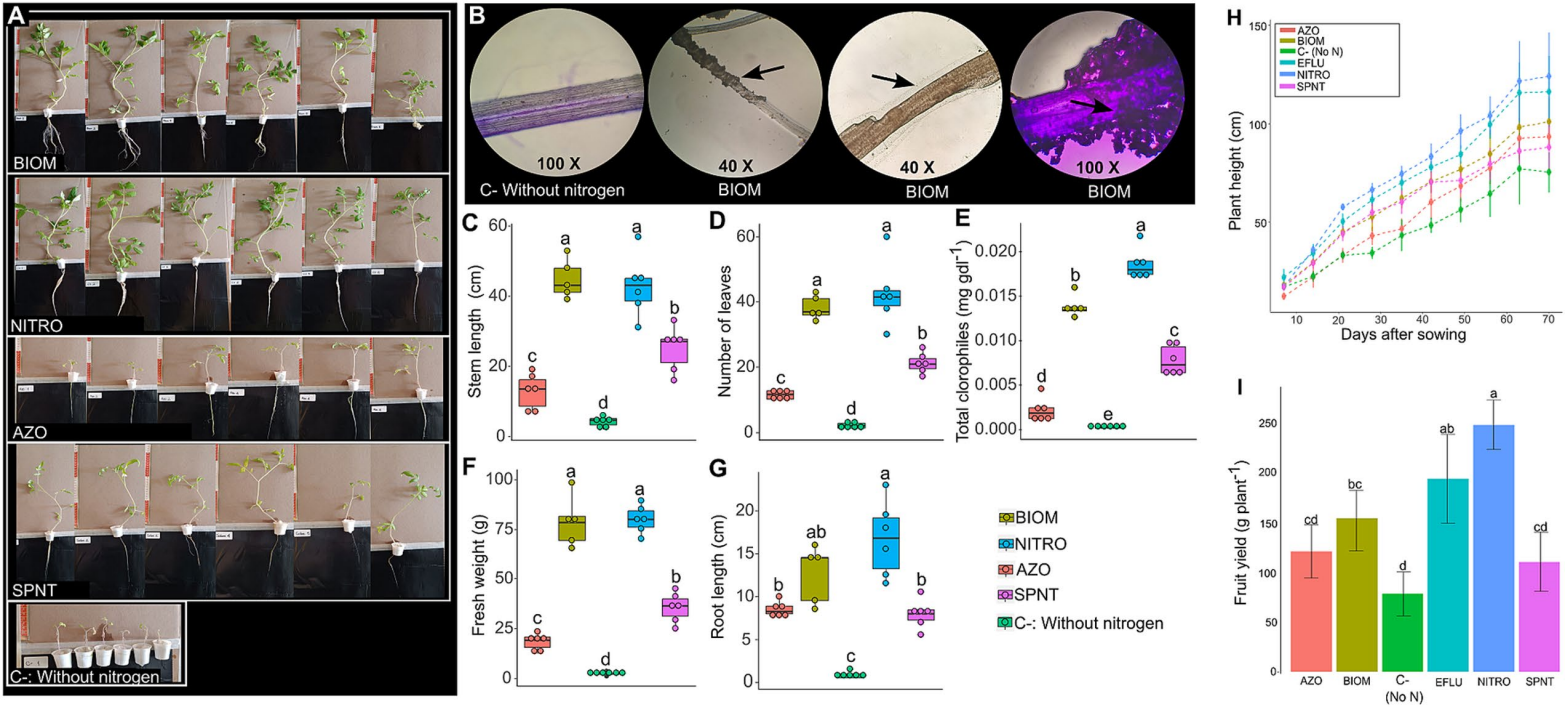
Growth of tomato plants under hydroponic and greenhouse conditions. For hydroponics, measurements were taken 45 days after sowing. **(A)** Photographs of the plants of each treatment. **(B)** Microscopic photographs of roots of tomato plants of the negative control without nitrogen supply and plants inoculated with the biomass resuspended in saline solution. **(C)** Stem length. **(D)** Number of leaves. **(E)** Total chlorophyll content. **(F)** Fresh weight. **(G)** Root length. For the greenhouse experiment, plant height **(H)** was followed from day 0 until day 70 after transplanting, and fresh weight yield **(I)** was measured by collecting tomato fruits between 45 and 90 days after sowing (DAS). Different letters indicate statistically significant differences (ANOVA-Tukey, *p* < 0.05).

Tomato plants cultivated under hydroponic conditions showed that the addition of microbial biomass to the nutrient solution resulted in plant growth like that obtained with nitrogen fertilization (Figure 4A). The use of a commercial product containing *Azotobacter* sp. and *Azospirillum* sp., while promoted significantly greater growth than the negative control (C−), did not reach the level of the positive control with nitrogen (NITRO) (Figure 4A). In contrast, plants from the AZO and SPNT treatments exhibited poor foliar development, limited root growth, and chlorosis symptoms. Plants in the negative control (C−) only developed a pair of true leaves (Figure 4A), highlighting the importance of nitrogen for plant development. Figure 4B shows microscopic images of tomato roots from the BIOM and C − treatments, where biofilm formation on the root surface is visible in the BIOM treatment. Figures 4C–G quantitatively show that the BIOM treatment produces values statistically equivalent to those obtained with nitrogen fertilization and significantly higher than the negative control without nitrogen input.

The same treatments described above were applied to tomato plants grown in a greenhouse. An additional treatment, EFLU, consisted of directly using the effluent from the bioreactor. The results show that direct application of the effluent (EFLU) resulted in plant growth and yield comparable to that obtained with nitrogen fertilization (NITRO) (Figure 4H). Although the use of biomass (BIOM) led to significantly greater growth and yield than the negative control, it did not reach the levels achieved with nitrogen fertilization (NITRO) (Figure 4I). The AZO and SPNT treatments showed growth and yield comparable to those of the negative control (Figure 4I).

### 3.4 The taxonomic composition of the SANF community reveals key taxa involved in nitrogen fixation and plant growth promotion

**Figure 5:**
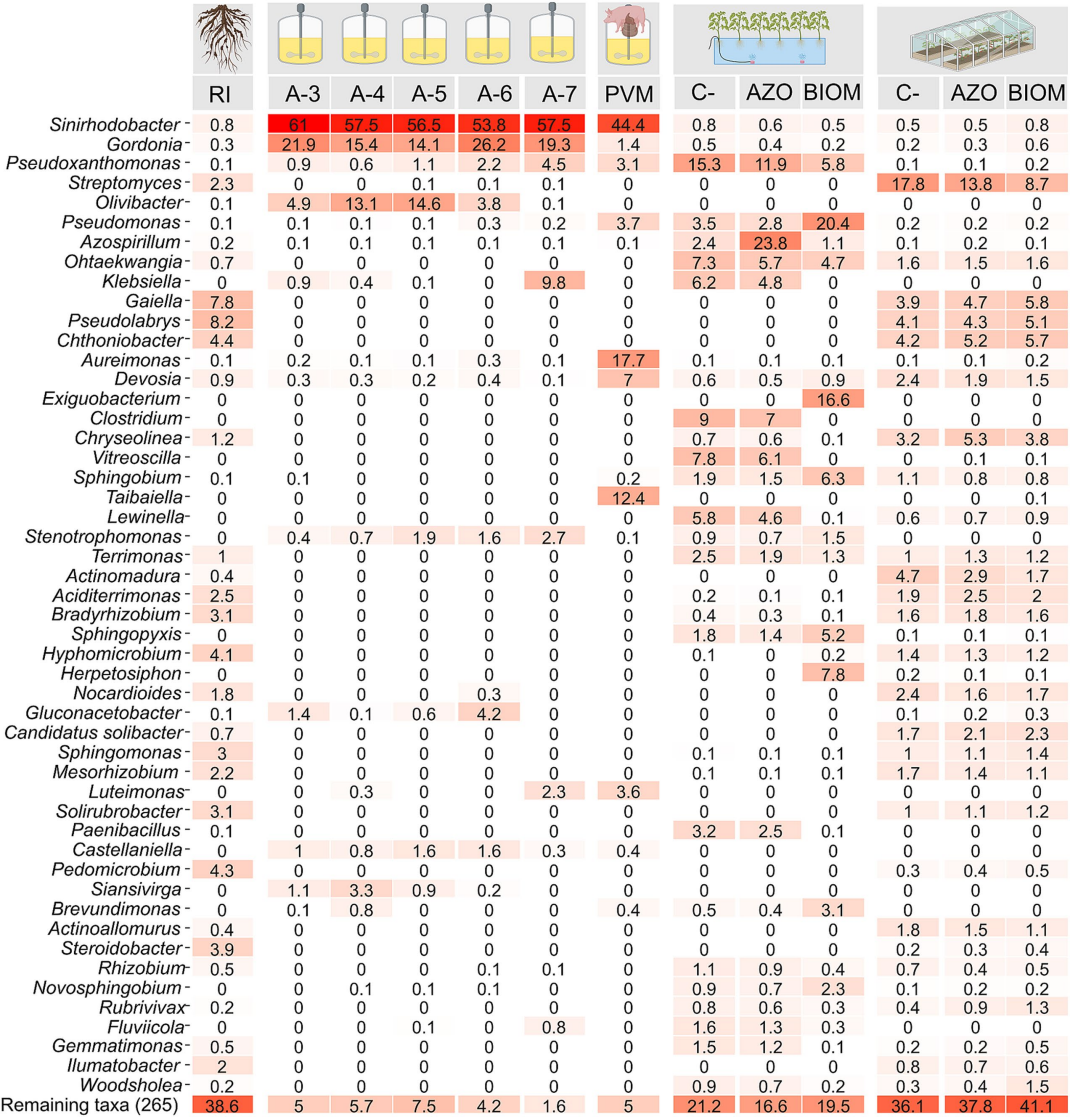
Heatmap showing the relative abundance values for the 30 most abundant genera in each analyzed treatment. RI: rhizospheric inoculum; PVM: self-assembled community fed with a mixture of volatile fatty acids (VFAs) derived from pig manure. C-: negative control without nitrogen input. AZO: microbial community from the rhizosphere of tomato plants inoculated with a commercial product based on *Azotobacter chroococcum* and *Azospirillum* sp. BIOM: microbial community from the rhizosphere of tomato plants inoculated with the microbial biomass of the nitrogen-fixing community fed with a mixture of VFAs derived from pig manure.

DNA samples were collected at different stages of the experimental setup for bacterial taxonomic profiling. At the end of the selection phase, a similar taxonomic composition was observed across all dilutions, with *Shinirhodobacter* spp. being the most abundant taxon, followed by *Gordonia* spp. (Figure 5). In the community obtained from the V-6 reactors, *Shinirhodobacter* spp., *Gordonia* spp., and *Gluconoacetobacter* spp. together accounted for 84.2% of the total relative abundance. When the self-assembled community from the V-6 dilution was fed with a mixture of VFAs derived from pig manure, *Shinirhodobacter* spp. remained the most abundant taxon; however, *Gordonia* spp. and *Gluconoacetobacter* spp. tended to disappear, while *Aureimonas* spp. and *Taibaiella* spp. increased in abundance (Figure 5). In the hydroponic experiment, plants in the C- treatment were not inoculated; therefore, their rhizosphere composition reflected endophytic microorganisms from the seeds or microbes originating from the irrigation water. The taxonomic composition of C- plants was very similar to that of the AZO treatment, with the difference that *Azospirillum* sp. (23.8%) increased in abundance, whereas *Azotobacter* sp. was not detected. In BIOM-treated plants, some taxa such as *Pseudomonas* spp., *Exiguobacterium* spp., *Sphingobium* spp., *Herpetosiphon* spp., and *Pseudoxanthomonas* spp. increased in relative abundance, even though some of them were present at very low concentrations in the SANF community and were initially undetectable. Conversely, in the BIOM treatment, taxa such as *Clostridium* spp., *Lewinella* spp., and *Vitreoscilla* spp. decreased in relative abundance. In the greenhouse experiment, the taxonomic composition of plants in the C-, AZO, and BIOM treatments did not show substantial changes.

**Figure 6:**
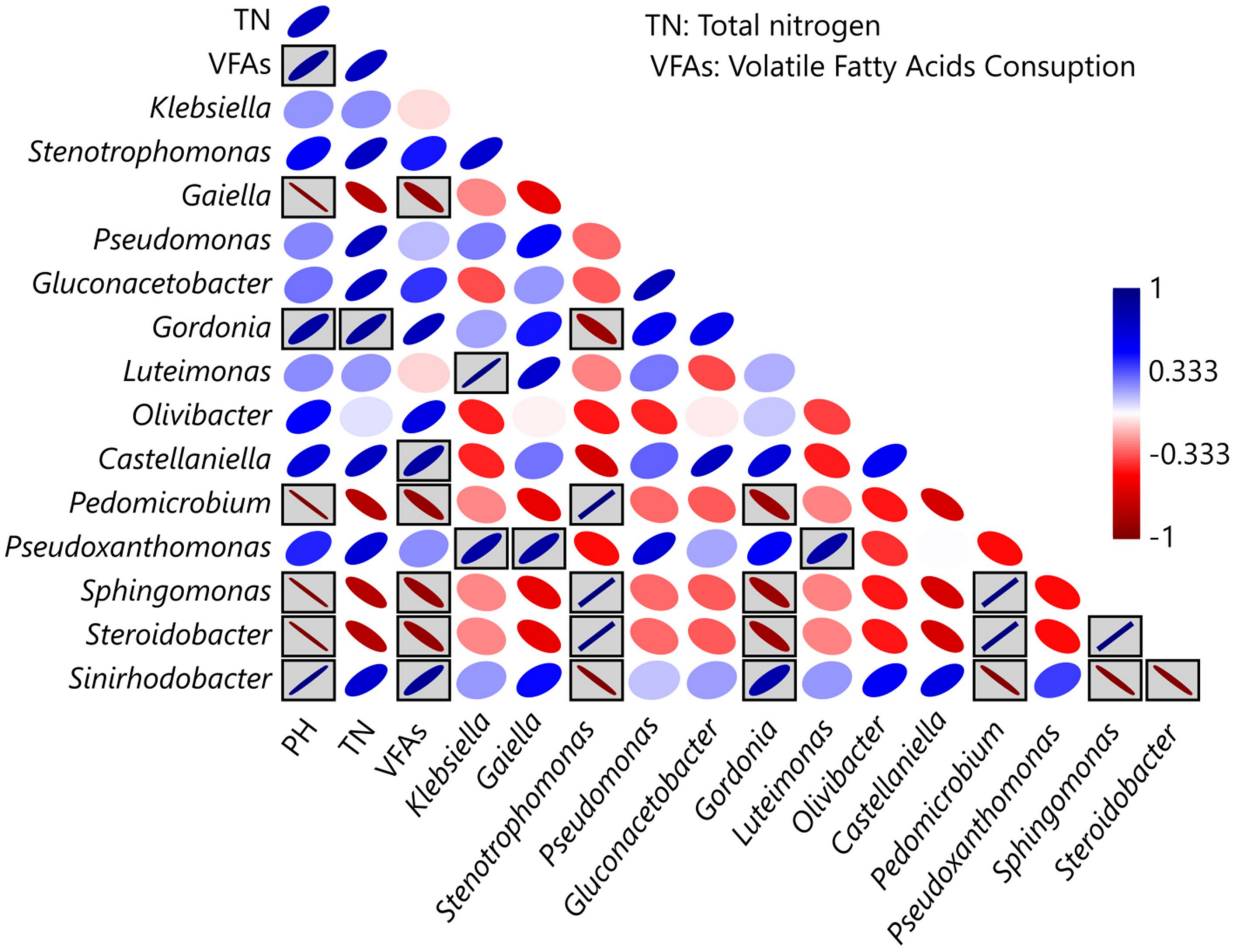
Pearson correlation between physicochemical parameters and the relative abundance of the dominant microbial taxa identified in the selection phase. The strength and direction of correlations are represented by ellipse orientation and color intensity. Gray-shaded boxes highlight correlations that are statistically significant (*p* < 0.05), while no boxes denote non-significant relationships.

During the selection phase, Pearson correlation analysis revealed significant associations between physicochemical parameters and the relative abundance of the dominant taxa (Figure 6). Both positive and negative correlations were detected, indicating that different microbial groups responded in contrasting ways to environmental gradients within the bioreactors. Statistically significant correlations (*p* < 0.05) are highlighted by ellipses within gray-shaded boxes.

## 4 Discussion

This study demonstrates the feasibility of engineering a self-assembled nitrogen-fixing community (SANF) which can utilize volatile fatty acids (VFAs) as the primary carbon source, and promote plant growth at levels comparable to those achieved with synthetic nitrogen fertilization. Instead of isolating or engineering a single diazotroph, we allowed microbial communities to self-organize under nitrogen- and carbon-limited conditions with VFAs as selective substrates. This process yielded specialized consortia with emergent properties, including enhanced nitrogen accumulation, efficient root colonization, and improved plant growth promotion. Importantly, we observed that when introduced into tomato plants, these consortia successfully colonized roots and delivered significant growth benefits, whereas a traditional *Azotobacter* sp. and *Azospirillum* sp. failed to establish or provide comparable effects.

During the ME process, butyric and propionic acids were preferred during the selection phase. In contrast, acetic acid showed lower utilization, which may reflect differences in metabolic preferences among the dominant taxa. Interestingly, despite continuous aeration in the bioreactors, the SANF maintained nitrogen fixation, implying that taxa within the consortium likely employed oxygen-protective strategies such as microaerophily, biofilm formation, or respiratory protection, which are common among free-living diazotrophs (Zuberer, 2021). Future culture-dependent and acetylene reduction tests could confirm whether these taxa actively fix nitrogen or facilitate BNF indirectly through supportive metabolic roles, such as scavenging oxygen or supplying essential cofactors.

In the selection phase, the V-6 community exhibited the highest nitrogen fixation; however, it was not the greatest consumer of VFAs, suggesting that it is more efficient in utilizing carbon sources. In terms of taxonomic composition, the dominance of *Sinirhodobacter* spp. and *Gordonia* spp., genera rarely associated with nitrogen fixation, suggests that VFAs act as ecological filters promoting alternative diazotrophic assemblages. Unlike the other communities, the V-6 community showed a higher abundance of *Gluconacetobacter* spp., which may play an important role in VFA consumption and/or nitrogen fixation. These interactions are consistent with the concept of functional convergence, where distinct taxa converge on a shared ecological function under selective pressures (Estrela et al., 2022). The correlation analysis suggests that *Sinirhodobacter* is associated with VFA consumption, whereas *Gordonia* is associated with nitrogen accumulation. This indicates a collaborative interaction in which one bacterium transforms carbon sources into high-energy metabolic intermediates, while the other carries out nitrogen fixation. This hypothesis could be tested using metagenomic or metatranscriptomic approaches.

Although the most abundant taxa in these communities are not all classical diazotrophs, their proliferation under nitrogen-depleted conditions strongly suggests ecological dependence on nitrogen fixed by a subset of the microbial community. This is consistent with ecological principles observed in plant systems, where a few diazotrophs sustain broader microbial or plant-associated networks (Zhang et al., 2022; Li et al., 2025). In such consortia, high-abundance taxa may not fix nitrogen themselves, but may thrive due to efficient assimilation of biologically fixed nitrogen. In our opinion, the isolation of specific nitrogen-fixing bacteria would create an artificial community, which may have a more limited adaptability and less resilience to environmental stressors.

We performed a functional prediction using PICRUSt2 focusing on genes related to nitrogen fixation. The results (Supplementary Figure S3) indicate a potential presence of both nif and vnf genes throughout the process. Interestingly, genes associated with alternative nitrogenases (*vnfD*, *vnfH*, and *vnfK*) were the most abundant. The coexistence of both nitrogenase systems raises the question of which one is preferentially utilized under the given conditions. While the Mo-dependent nitrogenase (nif) is generally more energy-efficient and widespread among diazotrophs (Pi et al., 2022), the higher abundance of vnf genes may reflect an adaptive advantage under molybdenum-limited or more reducing conditions, where vanadium nitrogenase can operate more effectively. Moreover, alternative nitrogenases are often associated with anaerobic or microaerophilic communities (Cuevas-Zuviría et al., 2024). These findings suggest that microbial communities may modulate the use of different nitrogenase systems in response to trace metal availability and oxygen gradients. However, it is important to note that these are only functional predictions, and further validation using shotgun metagenomics or metatranscriptomics will be necessary to confirm the presence and activity of these genes.

As expected, increasing dilution levels resulted in a decrease in diversity due to the applied ecological selection conditions. However, the same taxa remained the most abundant, indicating that community assembly in this case follows a deterministic pattern. Recent studies have described this type of assembly as an example of functional convergence, reflecting metabolic self-organization as an emergent property of microbial communities (Estrela et al., 2022; Chang et al., 2023). Here, nutrient availability and environmental constraints drive the emergence of simplified but functionally specialized consortia. This pattern has been described for other engineered communities (Kang et al., 2020; Díaz-García et al., 2021) and reflects an ecological trade-off: reduced taxonomic diversity may decrease overall resilience but enhance functional efficiency by minimizing competitive interactions. The replacement of *Gordonia* spp. and *Gluconoacetobacter* spp. by *Aureimonas* spp. and *Taibaiella* spp. after exposure to waste-derived VFAs suggests a functional shift in the community toward taxa better adapted to complex organic acid mixtures.

The SANF community exhibited robust growth and nitrogen fixation when fed with a VFA mixture derived from pig manure, achieving over 90% VFA consumption and a peak in nitrogen accumulation during the stationary phase. This is the first evidence, to our knowledge, of a nitrogen-fixing consortium effectively utilizing waste-derived VFAs as the sole carbon source. A key ecological feature of the SANF was its apparent extracellular nitrogen release, reaching levels up to 12 mg/L. Although energetically costly and generally suppressed in diazotrophs (Batista et al., 2021), extracellular nitrogen release has been reported in certain free-living diazotrophs (Ortiz-Marquez et al., 2012; Ambrosio et al., 2017) and may represent a cooperative trait, promoted by carbon abundance or community-level regulation. This finding is particularly relevant from an ecological and biotechnological perspective, as it suggests that microbial consortia can act as nitrogen biofactories, providing a direct nitrogen source for plants or other bioprocesses, potentially reducing dependence on synthetic fertilizers. The observed increase in total nitrogen concentration in the culture medium, reaching up to 12.7 mg·L^−1^, was obtained under strictly controlled, nitrogen-limited conditions. The bioreactors were operated as closed systems with respect to reactive nitrogen, where the only nitrogen input was atmospheric N₂ supplied via controlled air injection. No exogenous sources of fixed nitrogen (e.g., ammonium, nitrate, amino acids) were added during the enrichment process.

Recent efforts have focused on isolating individual VFAs from mixed VFA streams to evaluate their potential as carbon sources for different bioprocesses (Sun et al., 2024). However, the separation of individual VFAs is inefficient and costly (Atasoy et al., 2018). In this context, valorization strategies that use mixed VFAs may be more feasible and applicable. Our results show that the mixed VFA substrate performed better in terms of nitrogen fixation than using acetic acid or propionic acid individually. It is important to note that, during the distillation process used for VFA recovery, other undetected compounds may have been present and could have contributed to the growth of the self-assembled community. Although waste-derived VFAs are not irreplaceable, they provide a practical and sustainable alternative to conventional substrates. Their application reduces the reliance on synthetic carbon sources and supports environmental management of organic waste. From a biotechnological standpoint, this strategy aligns with principles of sustainability and circularity in agricultural systems.

In both hydroponic and soil systems, inoculation of tomato plants with the self-assembled community enhanced growth and yield to levels comparable to conventional chemical fertilization. In the BIOM hydroponic treatment, certain taxa such as *Pseudomonas* spp. (20.7%) and *Sphingobium* spp. (6.8%) were enriched, while *Exiguobacterium* spp. (11.9%) emerged despite being undetectable in the original inoculum, suggesting recruitment from low-abundance populations. This highlights that inoculating plants with a diverse microbial community provides the opportunity to recruit the most effective taxa, demonstrating plasticity and adaptation to the tomato rhizosphere despite being enriched under non-rhizosphere conditions and with carbon sources uncommon in plant-associated environments. This adaptability reflects the consortium’s broad metabolic versatility, a key trait for successful establishment in field conditions where competition with native microbial communities is a major bottleneck for biofertilizers (Roy, 2021). While *Pseudomonas* spp. is known to fix nitrogen, *Exiguobacterium* spp. and *Sphingobium* spp. are primarily recognized for plant growth promotion through mechanisms such as indole-3-acetic acid production, phosphate solubilization, siderophore formation and biological control (Huang et al., 2024; Sur and Sathiavelu, 2024; Wang et al., 2025). Additionally, some taxa abundant in controls, such as *Clostridium* spp., *Pseudoxanthomonas* spp., and *Paenibacillus* spp., decreased in BIOM, suggesting that growth promotion is likely driven by multiple ecological interactions rather than nitrogen fixation alone, and possibly a suppression effect is also involved. Interestingly, in the AZO treatment, *Azotobacter* sp. was absent, and although *Azospirillum* sp. reached 24.7% of relative abundance in hydroponics, nitrogen fixation was insufficient to sustain normal plant growth. This may be related to its microaerophilic metabolism (Alexandre, 2015), which is unsuitable for hydroponic systems with high dissolved oxygen.

In the greenhouse experiment, unlike the hydroponic trial, the BIOM treatment was not sufficient to achieve yields comparable to the NITRO treatment. This may be due to the low inoculum dose applied, as no substantial changes in taxonomic composition were observed in inoculated plants. Additionally, the characterization of the rhizosphere microbiome may have been conducted too early; sampling at later plant growth stages might have allowed sufficient time for the inoculated microbiome to establish in the rhizosphere. A similar study reported that inoculation with the microbial biomass of a nitrogen-fixing self-assembled community was able to reconfigure the rhizosphere microbiome and improve plant growth to levels comparable to those achieved with conventional nitrogen fertilization (Gutiérrez et al., 2022). The direct use of effluent outperformed the centrifuged biomass, likely because of the residual nutrients and fermentation-derived metabolites it contained. The superior plant growth observed with the direct application of the bioreactor effluent highlights the importance of maintaining both living microbial consortia and fermentation-derived metabolites. These metabolites may function as signaling molecules or additional nutrient sources, promoting rapid plant and microbial responses. From an ecological perspective, this approach better mimics natural systems, where plants interact with complex networks of microorganisms and metabolites rather than with isolated strains.

Overall, this study provides strong evidence that microbiome engineering (ME) can leverage ecological principles of community assembly and cooperation to develop effective nitrogen-fixing biofertilizers. By integrating waste-derived carbon sources, this approach also aligns with circular economy strategies, converting agro-industrial residues into functional microbial communities capable of sustaining biological nitrogen fixation in soil ecosystems. However, several limitations should be acknowledged. The low inoculum dose and early rhizosphere sampling in the greenhouse may have underestimated the ability of the self-assembled community to establish and modulate the native microbiome. Moreover, the reliance on 16S rRNA sequencing limited the detection of low-abundance or functionally relevant taxa. More targeted or integrative approaches, such as *nifH* amplicon sequencing, metagenomic and metatranscriptomic analyses, quantitative PCR of nitrogen fixation genes, and functional assays like the acetylene reduction assay (ARA) or ^15N₂ incorporation, would allow a more accurate characterization of diazotroph diversity and activity. In hydroponics, for the SPNT treatment, nitrogen availability was inferred from total nitrogen content without distinguishing between inorganic and organic forms, and the potential effects of residual VFAs or fermentation-derived metabolites on plant growth remain unclear.

Two main future research directions emerge from this work. First, focusing on the stability of the self-assembled community in bioreactors could enhance nitrogen fixation efficiency and allow the production of bioavailable nitrogen forms as a direct product. Second, efforts could focus on maximizing microbial biomass production for its use as a biofertilizer. In both cases, identifying key taxa through culturomics and other omics approaches will be crucial to designing a more simplified yet stable community that retains the same functional traits.

## 5 Conclusion

This study shows the feasibility of microbiome engineering (ME) as a novel strategy for developing effective, low-cost biofertilizers based on self-assembled microbial communities. By using volatile fatty acids (VFAs) derived from animal waste as the sole carbon source, we enriched a nitrogen-fixing consortium capable of releasing bioavailable nitrogen and promoting plant growth under both soil and hydroponic conditions. The selected community showed adaptability, functional stability, and the potential to reshape the rhizosphere in favor of beneficial taxa. These findings highlight the ecological and technological advantages of designing community-level biofertilizers over traditional single-strain products. Moreover, the integration of waste valorization with microbial biotechnology offers a scalable solution aligned with circular economy principles. Future research should focus on field validation, long-term community stability, and the optimization of biofertilizer formulations to ensure robust performance across diverse agricultural systems.

## Data Availability

The datasets generated and analyzed for this study are not publicly available because they are under patent registration. However, they can be made available by the authors upon reasonable request. The DNA raw sequences for this project were deposited at the National Center for Biotechnology Information (NCBI) under Bioproject PRJNA1297193.

## Footnotes

^1^ https://qiime2.org/
