# Supplementary material for: Engineering nitrogen-fixing microbiomes with waste-derived carbon sources: toward circular and resilient biofertilizer solution
Source: Front Microbiol. 2025 Sep 23;16:1676616. doi: 10.3389/fmicb.2025.1676616 (PMC12500719; doi:10.3389/fmicb.2025.1676616)
Supplement: Supplementary file 1 [file Table_1.docx]

***Supplementary Material***

Engineering Nitrogen-Fixing Microbiomes with Waste-Derived Carbon Sources: Toward Circular and Resilient Biofertilizer Solution

**Nicolás Rodríguez-Romero^1-2^, Juan Carlos Clavijo-Salinas^2^, Julien Wist ^2,3^, Carlos Gutierrez^2^, Daniel Uribe-Velez^1^, Elaine Holmes ^3,4,5^, Janeth Sanabria ^3*^**

^1^Agricultural Microbiology Research Group, Biotechnology Institute, National University of Colombia, Bogotá, Colombia.

^2^Environmental Microbiology and Biotechnology Laboratory, School of Natural Resources and Environmental Engineering, Faculty of Engineering, Universidad del Valle, Cali, Colombia.

^3^Australian National Phenome Centre and Centre for Computational and Systems Medicine, Health Futures Institute, Murdoch University, Harry Perkins Building, Perth, WA 6150, Australia

^4^Division of Digestive Diseases, Faculty of Medicine, Imperial College, London W2 1NY, UK

^5^Institute of Global Health Innovation, Imperial College London, London, SW7 2AZ, UK^3^

*** Correspondence:**Corresponding Author
janneth.sanabriagomez@murdoch.edu.au

**Keywords: bioeconomy, waste valorization, targeted bioprospecting, self-assembled community, nitrogen.
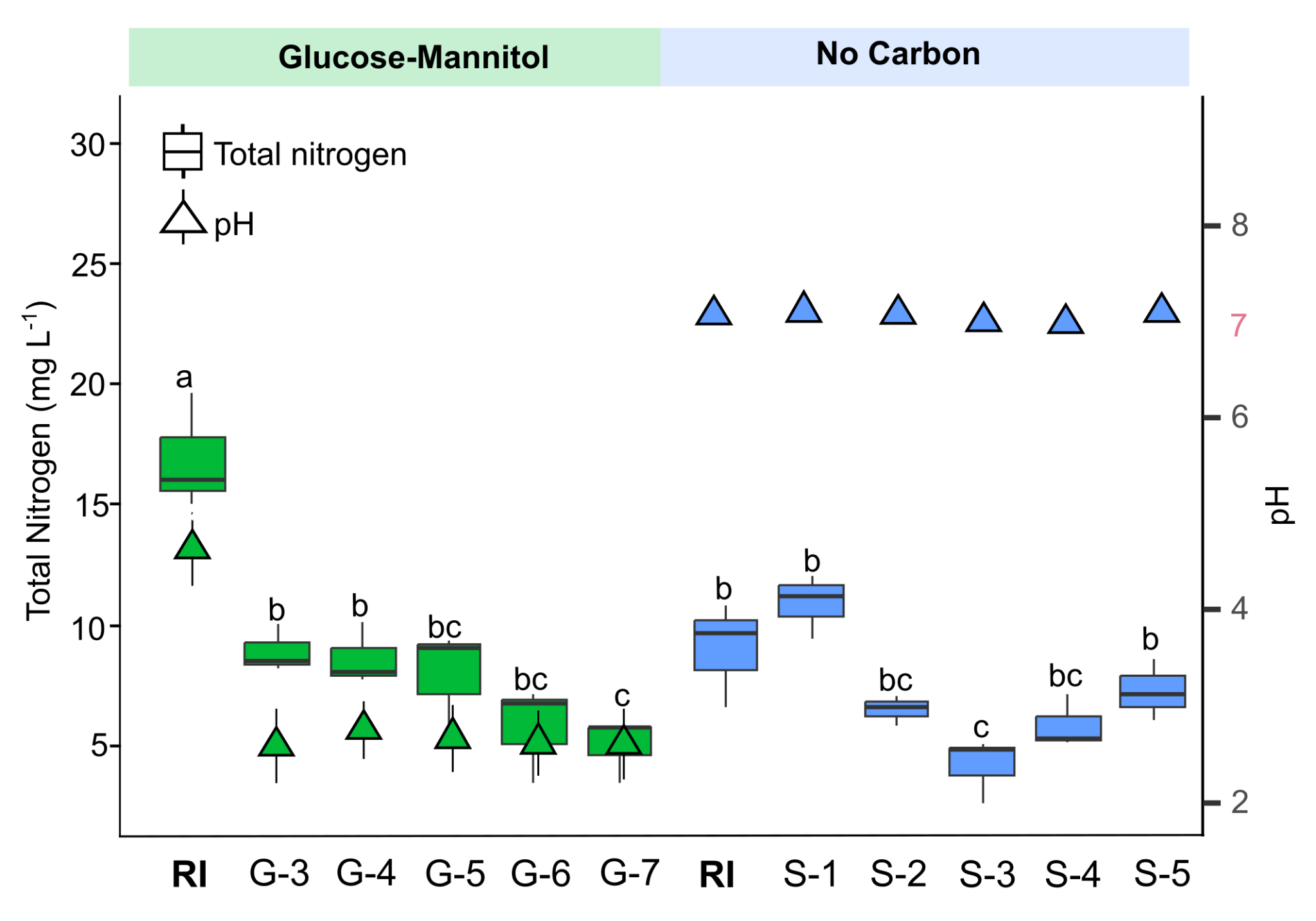
**

**Supplementary Figure 1.** Chemical variables for the Glucose-mannitol and no-carbon bioreactors in the selection phase. Total nitrogen (boxes) and pH (triangles) for each of the dilutions. n =3. RI: rhizosphere inoculum. Different letters indicate significant differences in total nitrogen (ANOVA-Tukey, p < 0.05).

**
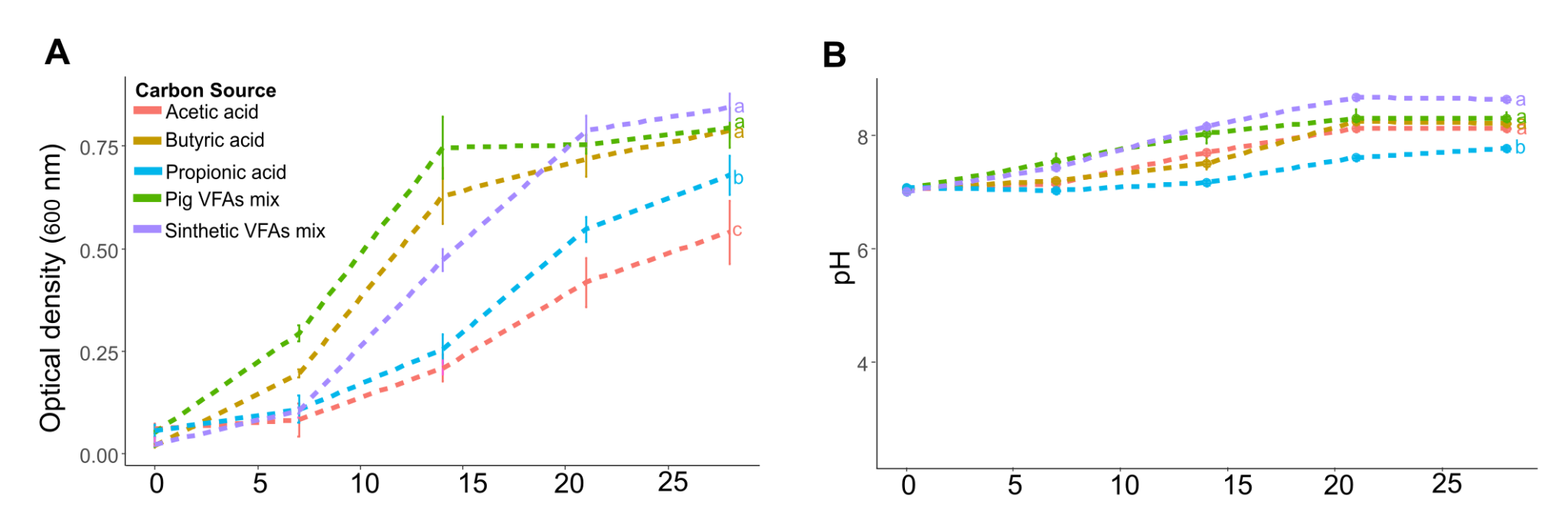
**

**Supplementary Figure 2.** Microbial growth of bioreactors fed with a VFAs mix from pig manure. Optical density (A) and pH (B) from day 0 to 28. VFAs: volatile fatty acids. n = 3.

**
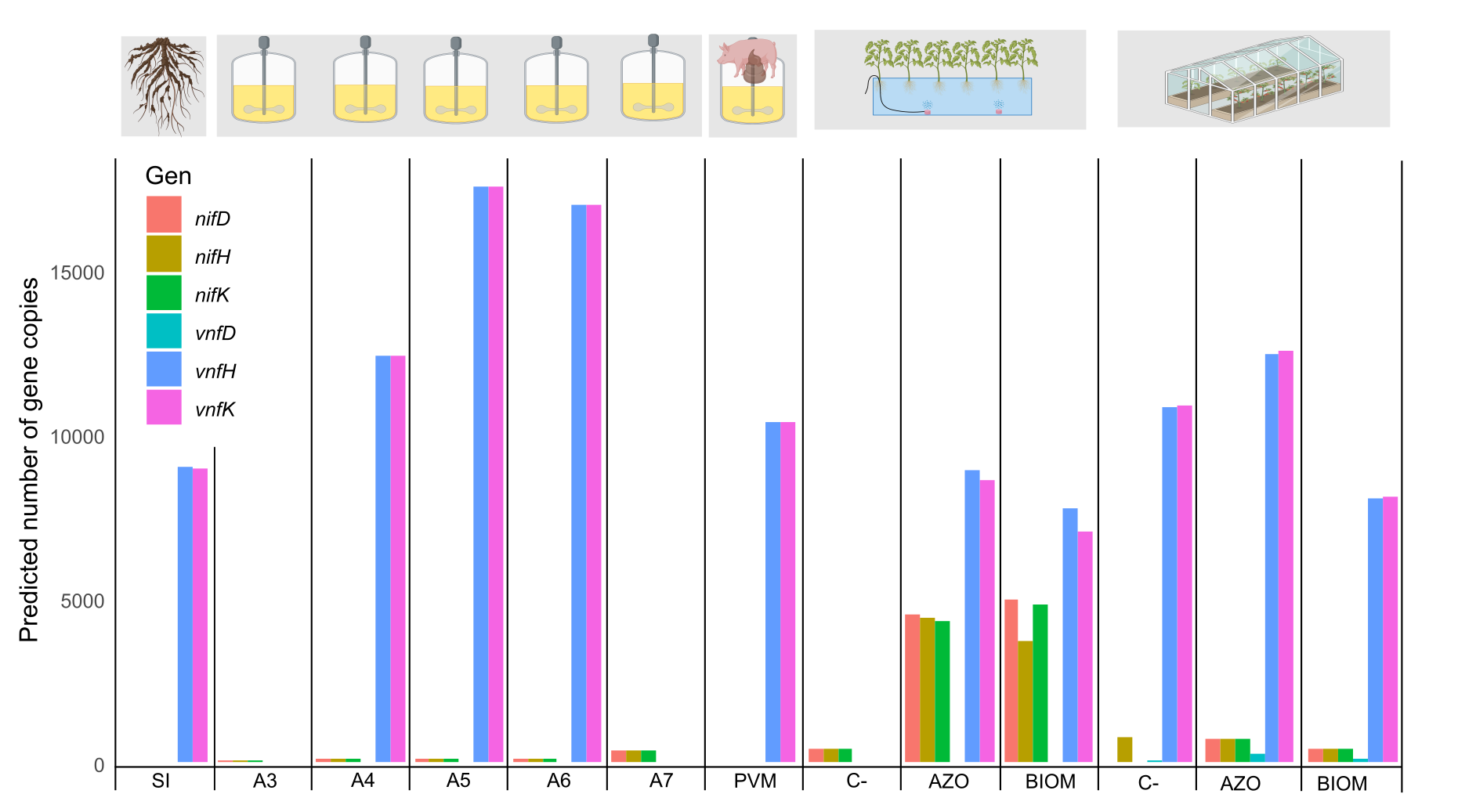
**

**Supplementary Figure 3**. Predicted functional profiles of nitrogenase-related genes (nifD, nifH, nifK, vnfD, vnfH, and vnfK) obtained with PICRUSt2 based on 16S rRNA gene sequences across different samples and experimental conditions. Bars represent the predicted number of gene copies in each treatment,
